# 

**Published:** 1887-06

**Authors:** 


SUBSCRIPTION PRICE, $3,00 PER YEAR, IN ADVANCE.

Whole Number, 330 JUNE, 1887. Vol. LIV., Number 6.

### THE

### CHICAGO MEDICAL JOURNAL AND EXAMINER

Editor: S. J. JONES, M.D., LL.D.

PUBLISHED MONTHLY BY

THE CLARK & LONGLEY COMPANY,

Under direction of

THE CHICAGO MEDICAL PRESS ASSOCIATION,

308-316 Dearborn Street.

